# Whipple’s Disease, a Rare Cause of Constrictive Myopericarditis and Right-Sided Heart Failure

**DOI:** 10.7759/cureus.99934

**Published:** 2025-12-23

**Authors:** Juliette Marot, Sophie Pierard, Laurence Bamps, Halil Yildiz, Jean Cyr Yombi

**Affiliations:** 1 Infectious Disease, Centre Hospitalier Universitaire, Université Catholique de Louvain, Namur, BEL; 2 Cardiology, Centre Hospitalier Universitaire, Université Catholique de Louvain, Brussels, BEL; 3 Internal Medicine and Infectious Disease, Cliniques Universitaires Saint-Luc, Université Catholique de Louvain, Brussels, BEL

**Keywords:** constrictive pericarditis, heart failure, iris, tropheryma whipplei, whipple's disease

## Abstract

Whipple’s disease is a rare, chronic multisystemic infection caused by *Tropheryma whipplei*. Although the disease typically presents with arthritis, diarrhea, fever, and lymphadenopathy, it may also involve the heart. Cardiac involvement is uncommon but may include constrictive myopericarditis, a potentially life-threatening cause of right-sided heart failure. We report the case of a 67-year-old woman admitted with decompensated right-sided heart failure. Echocardiography and chest CT revealed constrictive pericarditis with an intrapericardial mass initially suspicious for malignancy. Duodenal and pericardial biopsies demonstrated Periodic acid-Schiff (PAS)-positive macrophages, and polymerase chain reaction (PCR) confirmed *T. whipplei* infection. The patient had a history of chronic seronegative arthritis and later developed diarrhea and severe malnutrition, consistent with Whipple’s disease. Standard antibiotic therapy was initiated with intravenous ceftriaxone, followed by trimethoprim-sulfamethoxazole. Her course was complicated by suspected immune reconstitution inflammatory syndrome, ongoing inflammation, and nutritional decline, rendering surgical pericardiectomy unsafe. A trial of oral corticosteroids in addition to antibiotics resulted in clinical improvement and significant regression of the pericardial mass. This case highlights an unusual cardiac manifestation of Whipple’s disease and emphasizes the importance of considering this diagnosis in patients with unexplained constrictive pericarditis and systemic symptoms. To our knowledge, this is the first reported case of constrictive myopericarditis due to Whipple’s disease successfully managed without pericardectomy. Our report underscores the potential role of a conservative medical approach in frail or high-risk patients and illustrates the need for a better understanding of immunopathogenesis to guide therapeutic strategies.

## Introduction

Whipple’s disease is a rare, chronic, multisystem infectious disorder caused by the gram-positive bacillus *Tropheryma whipplei*. The disease was first described in 1907; however, the bacterium was not successfully cultured until 2000 [1]. Initially named *Tropheryma whippelii*, from the Greek *trophi* (nourishment) and eryma (barrier), the organism’s name reflects the malabsorption frequently observed in affected patients [2]. 

Although *T. whipplei* is relatively common in the environment and among human hosts, transmitted primarily via the orofecal route, Whipple’s disease remains exceedingly rare, with an estimated incidence of one per 1,000,000 individuals [3]. Asymptomatic carriage is well documented, and most individuals exposed to *T. whipplei* do not develop clinical disease, likely due to effective innate and adaptive immune responses. In contrast, a small subset of individuals, possibly due to genetic susceptibility, progress to chronic systemic illness. Notably, human leukocyte antigen (HLA) alleles such as HLA-DRB113 and HLA-DQB106 have been associated with increased susceptibility to disease progression [4]. 

Confirmatory diagnosis typically requires at least two of the following three criteria: presence of periodic acid-Schiff (PAS)-positive macrophages (first described by Black-Schaffer in 1949 [5]), positive *T. whipplei* polymerase chain reaction (PCR), or immunohistochemical staining [4,6]. However, PAS-positive macrophages are not specific and may also be observed in other conditions such as *Mycobacterium avium-intracellulare*, *Rhodococcus equi*, *Bacillus cereus*,* Corynebacterium* species, and *Histoplasma capsulatum* [7]. Moreover, only a limited number of serologic assays can distinguish classical Whipple’s disease from asymptomatic carriage [8]. 

Clinically, the disease most often presents with a characteristic tetrad of polyarthralgia, chronic diarrhea, fever, and lymphadenopathy [9]. While gastrointestinal involvement leading to malabsorption is the hallmark feature, multiple organ systems can be affected, including the central nervous system and, less commonly, the heart. This broad and sometimes atypical presentation often contributes to delayed diagnosis [10,11]. Although rare, cardiac involvement may manifest as constrictive myopericarditis, an uncommon but serious complication [12] 

## Case presentation

A 67-year-old woman was transferred to the internal medicine department in October 2024 after several months of complex hospital care. Her medical history included a low-grade chronic inflammatory syndrome accompanied by intermittent neutrophilia, nonspecific seronegative arthralgia, and unintentional weight loss.

In January 2024, polymyalgia rheumatica had been suspected after bilateral shoulder uptake on PET-CT, although a temporal artery biopsy was normal. A trial of corticotherapy initiated around March 2024 produced no clinical improvement. In February 2024, she also experienced an acute right popliteal artery occlusion treated with local thrombolysis and stenting, after which she was placed on dual antiplatelet therapy combined with a low dose of rivaroxaban. Over the following months, the patient’s condition progressively deteriorated, with worsening fatigue.

In July 2024, she developed grade III dyspnea, rapidly progressive lower-limb edema, and several days of non-bloody diarrhea, prompting admission to the cardiology department. Transthoracic echocardiography (TTE) revealed severely impaired right ventricular systolic function, reflected by a markedly reduced tricuspid annular plane systolic excursion (TAPSE) (8 mm), which is a direct measure of right ventricular systolic performance. Mild tricuspid regurgitation was present, and the estimated systolic pulmonary artery pressure was 34 mmHg, a value at the upper limit of normal. These findings raised suspicion of an infiltrative cardiac process. A bone scintigraphy showed no evidence of transthyretin amyloidosis. Serum protein electrophoresis demonstrated a purely inflammatory profile without monoclonal peaks, and urinalysis revealed no proteinuria. Decongestive therapy with continuous furosemide infusion and pleural drainage was initiated. Colchicine (1 mg/day) was introduced for pleuropericarditis despite fluctuating diarrhea, as non-steroidal anti-inflammatory drugs (NSAIDs) were contraindicated due to her dual antiplatelet therapy. Her course was further complicated by paroxysmal atrial fibrillation requiring amiodarone-assisted cardioversion and long-term anticoagulation (CHA₂DS₂-VASc score: 4).

Approximately one week later, she was transferred to internal medicine and gastroenterology for evaluation of worsening diarrhea, profound anorexia, and progressive weight loss. Iron-deficiency anemia was noted. Upper and lower endoscopies were performed, but biopsies were deferred due to dual antiplatelet therapy. Stool cultures, celiac serology, malabsorption studies, and endocrine tests were all negative. Her gastrointestinal condition continued to worsen, with variable bowel habits, intermittent hematochezia, complete anorexia, and rising inflammatory markers. A follow-up TTE again showed elevated filling pressures without a unifying diagnosis. After temporary withdrawal of antiplatelet therapy, repeat upper and lower endoscopy with biopsies was performed. Histopathology revealed no granulomas and no features of inflammatory bowel disease, but showed PAS-positive foamy macrophages; PCR confirmed *T. whipplei*, establishing the diagnosis of Whipple’s disease.

The patient was started on intravenous ceftriaxone (2 g/day) for 14 days. After an improvement, as inflammatory markers continued to rise and oxygen requirements increased, a nosocomial pulmonary superinfection was suspected, and treatment was escalated to meropenem for 14 days. She was then transitioned to oral trimethoprim-sulfamethoxazole (800/160 mg twice daily). Despite broad-spectrum antimicrobial therapy, the patient’s condition continued to decline, with a further increase in systemic inflammatory activity, raising suspicion for immune reconstitution inflammatory syndrome (IRIS).

On admission at our tertiary center at the end of October 2024, physical examination showed diffuse hematomas, marked jugular distension, positive hepatojugular reflux, and muffled heart sounds without murmurs. Respiratory examination revealed dyspnea at rest, orthopnea, and bibasilar hypoventilation, more pronounced on the right side, with oxygen saturation of 97% on 2 L supplemental oxygen. The abdomen was tense with shifting dullness, suggesting ascites. Severe pitting edema extended to the flanks and lower limbs. Neurological and joint examinations were normal. 

Laboratory tests showed marked inflammation and hematologic abnormalities: hemoglobin 9.8 g/dL (reference range, 12-16 g/dL), leukocytosis 21,000/mm³ (reference range, 4,000-10,000/mm³), thrombocytosis 550,000/mm³ (reference range, 150,000-450,000/mm³), CRP 135.4 mg/L (reference range, <5 mg/L), albumin 3.1 g/dL (reference range, 3.5-5.0 g/dL), alanine transaminase (ALT) 43U/L (reference range, <35 U/L), ferritin 312 ng/mL (reference range, 15-150 ng/mL), D-dimer 2,200 ng/mL (reference range, <500 ng/mL), and negative antinuclear antibody (ANA) and antineutrophil cytoplasmic antibodies (ANCA). Serum protein electrophoresis again showed a purely inflammatory polyclonal hypergammaglobulinemia. The κ/λ ratio was markedly abnormal at 7 (norm 0.26-1.65). Flow cytometry identified a small monotypic CD19⁺CD5⁻CD20⁺ κ-restricted B-cell population consistent with non-chronic lymphocytic leukemia (CLL) monoclonal B-cell lymphocytosis (0.053 × 10⁹/L). Given the κ/λ abnormality, a bone marrow biopsy was performed, which showed no evidence of malignancy.

**Figure 1:**
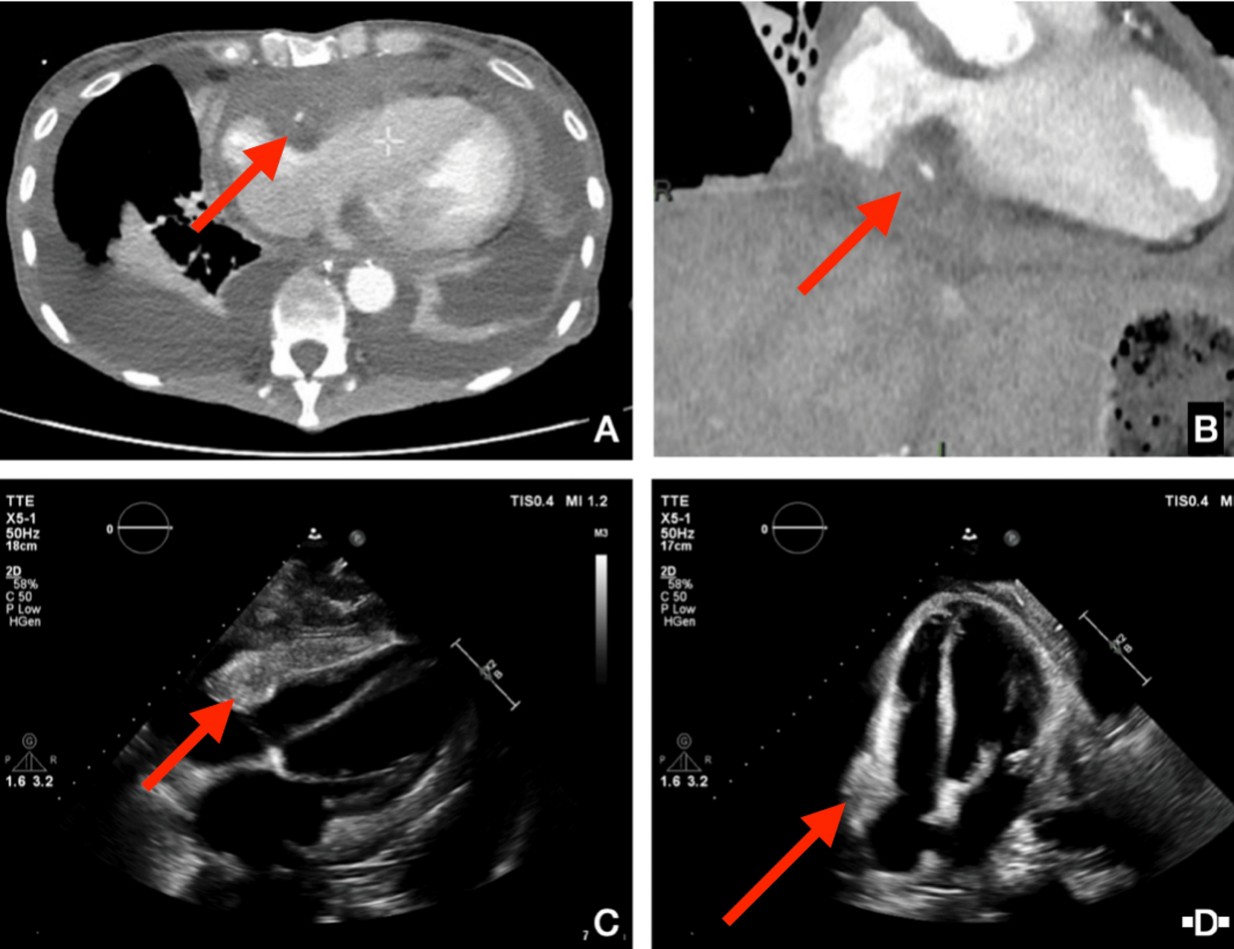
Imaging of constrictive pericarditis due to Whipple's disease A and B: Axial thoracic CT images, axial slice at the thoracoabdominal junction. The red arrows indicate a pericardial mass with mixed soft-tissue and fatty components encasing the right coronary artery. Additional signs of pericardial inflammation and bilateral pleural effusions are visible. Image B shows an enlarged view of the thickened pericardial tissue surrounding the coronary artery. C and D: Transthoracic echocardiography images demonstrating features of constrictive pericarditis. The red arrows highlight marked pericardial thickening (7 cm x 1.5 cm), particularly anterior to the right ventricle. Findings are consistent with a noncompliant pericardium restricting ventricular filling.

Because of increasing oxygen requirements and worsening dyspnea, a chest CT scan was obtained and unexpectedly revealed a large pericardial mass, initially suggestive of malignancy. Repeat echocardiography showed classic features of constrictive pericarditis, including a pronounced septal bounce, marked ventricular interdependence, and severely reduced lateral mitral annular motion (Figure 1). The mitral valve appeared slightly thickened but showed no mobile vegetations or features suggestive of infective endocarditis.

A PET-CT scan performed with cardiac preparation demonstrated a heterogeneous hypermetabolic reaction within the pericardial effusion and along the thoracic aorta, without evidence of valvular endocarditis. Additional findings included anasarca with mildly hypermetabolic diffuse subcutaneous infiltration, a large right pleural effusion, and a posterior-basal and intrafissural left pleural effusion, both non-hypermetabolic.

**Figure 2:**
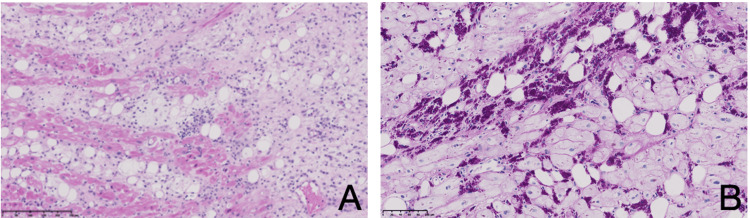
Histopathological findings of myocardial involvement in Whipple’s disease The myocardial samples shows an inflammatory infiltrate of mononuclear cells and neutrophils responsible for myocardial cellular lesions and an extensive involvement of the adipose and interstitial tissues of the myocardium by macrophages which contain granulations stained by periodic acid-Schiff (PAS). (A) Hematoxylin and Eosin staining, magnification x13; (B) Periodic-acid Schiff staining, magnification x21

A surgical pericardial and myocardial biopsy was obtained via mini-sternotomy. PCR was positive for *T. whipplei*, and histopathology confirmed Whipple’s disease involving both myocardium and pericardium (Figure 2), with acute myocarditis and fibrous pericarditis. PAS and PAS-diastase staining demonstrated macrophages filled with coarse PAS-positive granules. Congo red staining, performed to exclude amyloidosis, was negative. Because pericardial involvement can occur with indolent B-cell lymphomas such as marginal zone lymphoma, the biopsy was thoroughly examined for neoplastic infiltration. None was found. This presentation represents a true Whipple-associated myopericarditis.

Given her severe malnutrition, surgical pericardiectomy was contraindicated. She was treated with methylprednisolone (1 mg/kg/day), tapered by 4 mg every two weeks, together with continued oral trimethoprim-sulfamethoxazole. Two weeks later, she developed a retrosternal abscess (48 × 14 × 26 mm). All bacterial cultures and* T. whipplei* PCR were negative. In view of the aseptic presentation, corticosteroid therapy was increased by 4 mg, and the abscess was managed conservatively with wound packing, resulting in complete resolution in early 2025.

**Figure 3:**
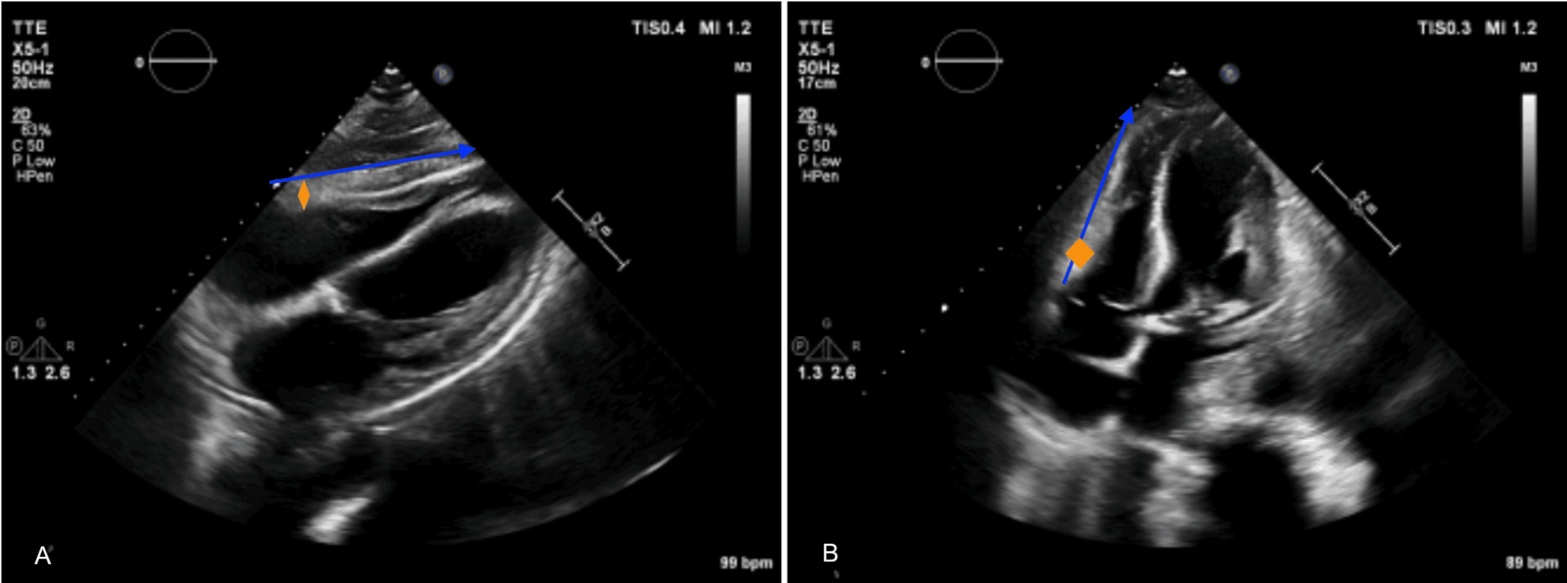
TTE after several weeks of corticosteroid therapy, still undergoing tapering regimen (A: subcostal view; B: Four-cavity view) Transthoracic echocardiography (TTE) shows regression of the pericardial mass, with a reduction in the compressive effect on the right ventricle and decreased pericardial thickening. Blue line = length 5.1 cm; orange line = width 0.7 cm (compared with 7 cm × 1.5 cm previously).

A major challenge throughout her course was profound protein-calorie malnutrition, exacerbated by corticosteroid therapy. Enteral nutrition and intensive physical therapy were initiated. Under treatment, her condition improved markedly. Echocardiography showed improved cardiac function and demonstrated regression of the pericardial mass, supporting an inflammatory process responsive to immunosuppression (Figure 3). 

Unfortunately, her known peripheral arterial disease complicated recovery. She developed stent thrombosis of the right lower limb following hip fracture surgery, facilitated by malnutrition and corticosteroids, necessitating transtibial amputation. 

After four months in acute care, the patient was transferred to a rehabilitation facility, where she remained for an additional six months. She was ultimately discharged in clinically stable condition, off corticosteroids, with long-term cotrimoxazole maintained for a planned 12-month course. From a Whipple’s disease perspective, her constrictive pericarditis improved, and no recurrence of diarrhea has been observed. 

## Discussion

Whipple’s disease presents with chronic diarrhea, weight loss, and migratory polyarthritis, yet atypical or incomplete presentations are increasingly recognized [6,7]. Profound asthenia and weight loss are common yet nonspecific findings [13,14]. In some patients, the disease may mimic a pseudotumoral syndrome before more typical signs appear [15]. 

Although cardiac manifestations have traditionally been considered rare, recent reports increasingly describe both pericardial and endocardial involvement, reflecting growing clinical awareness [16]. Myocardial disease remains less frequent. Historically, most cardiac cases were diagnosed postmortem [12,17], but improved diagnostic methods have facilitated earlier recognition. Stojan et al. reported six cases of constrictive pericarditis, illustrating this evolving trend [7]. Our literature review supports this observation and provides an updated synthesis of this rare cardiac manifestation (Table 1). 

**Table 1:** Reported cases of constrictive pericarditis due to Whipple’s disease PAS: periodic acid–Schiff; EM: electron microscopy; TMP-SMX: trimethoprim–sulfamethoxazole; PCR: polymerase chain reaction

Study	Age/Sex	Prodromal Symptoms	Delay to Diagnosis	Heart Failure Manifestation	Pericardial Histology	GI Histology	Treatment	Outcome
Stojan et al. [7]	68, M	Arthralgias, myalgias	3 years	Edema, weight gain, dyspnea	Polymerase chain reaction (PCR Whipple+), periodic acid–Schiff (PAS+)	PCR Whipple+, PAS+	Ceftriaxone, TMP-SMX, doxycycline, hydroxychloroquine, pleural decortication	Death
Lahoud et al. [11]	57, M	Fatigue, edema, orthopnea, migratory arthralgias	18 months	Edema, dyspnea, orthopnea	PCR Whipple+, PAS+	Not specified	Trimethoprim–sulfamethoxazole(TMP-SMX) and ceftriaxone, pericardiectomy	N/A
Sutherland et al. [13]	45, M	Anemia, weight loss	6 months	Shortness of breath, fluid overload, hypotension	Not specified	Duodenal PAS+, EM+	Ceftriaxone, TMP-SMX, pericardiectomy	Survived
Thornton et al. [14]	56, M	Weight loss, fatigue, cognitive impairment	4 years	Cachexia, lower extremity edema	PAS+, PCR 16S positive for T. whipplei	Negative	Penicillin G (IV, 14 days), cotrimoxazole, pericardial stripping	Survived
Iqbal et al. [16]	58, M	Arthralgia	2 years	Orthopnea, dyspnea, edema	PAS+ macrophages, electron microscopy (EM+)	Jejunal PAS+ histiocytes	Ceftriaxone, tetracycline, pericardiectomy	Survived
Vlietstra et al. [17]	63, M	Arthralgias, weight loss	8 years	Dyspnea, ascites	PAS+ histiocytes, EM+	Duodenal PAS+, EM+	Tetracycline, pericardiectomy	N/A
Crake et al. [18]	37, M	Chest pain, shortness of breath	No delay	Chest pain, shortness of breath	PAS+ macrophages	Duodenal PAS+	Pericardiectomy, tetracycline	Survived
Hansen et al. [19]	69, M	Chronic oligoarthritis	4 months	Dyspnea	PCR Whipple+	Negative	No treatment before death	Death
Freychet et al. [20]	61, M	Arthralgia, diarrhea	Not specified	Dyspnea	PAS+ macrophages, EM+	Jejunal negative	Pericardiectomy, tetracycline	N/A
Lochouarn et al. [21]	49, M	Weight loss, fever, arthralgias	25 years	Dyspnea	PAS+, immunohistochemistry+	Duodenal PAS+, immunochemistry+, PCR+	Doxycycline, TMP-SMX, hydroxychloroquine, pericardiectomy	Survived
Makol et al. [22]	56, M	Seronegative arthritis	20 years	Dyspnea, ascites, edema	PAS+ macrophages, T. whipplei PCR+	Jejunal PAS+ histiocytes	Ceftriaxone, TMP-SMX, pericardiectomy	Survived
Lie and Davis [23]	67, M	Anemia, edema, abdominal pain	12 months	Dyspnea, orthopnea, peripheral edema	PAS+ histiocytes	PAS+ histiocytes	Supportive, digitalis, vitamin B12	Death
Lie and Davis [23]	38, F	Diarrhea, anemia, fever, joint pains, peripheral neuritis, decubitus ulcers	12 months	Cachexia	PAS+ histiocytes	PAS+ histiocytes	Supportive, erythromycin	Death

Beyond the cases summarized in Table 1, primarily pericarditis with or without myocardial involvement [19,23] or endocardial involvement [11,14,19], there is growing evidence that cardiac involvement in Whipple’s disease is significantly broader than traditionally appreciated. 

*T. whipplei *endocarditis is now a well-established entity and remains the most frequently reported cardiac form [15,24], whereas myopericardial presentations are far less documented despite increasing recognition. McAllister and Fenoglio confirm this under-recognition: adhesive pericarditis was present in up to 80% of cases, valvular fibrosis in over half, and myocardial fibrosis with PAS-positive macrophages in approximately 10-11%, often without any cardiac symptoms ante mortem [12]. Several others reports describe granulomatous myocarditis, myopericarditis, and overlapping phenotypes, sometimes mimicking cardiac sarcoidosis, emphasizing the protean behavior of cardiac Whipple’s disease beyond valvular infection [25].

This review emphasizes the marked heterogeneity and diagnostic difficulty of Whipple’s disease with cardiac involvement. Importantly, in several cases, cardiac symptoms precede gastrointestinal manifestations, or digestive involvement may never occur [14,19]. This underscores the importance of considering Whipple’s disease in patients with unexplained right-sided heart failure, particularly when no clear etiology is identified [16,20].

Diagnostic delays are common. Lochoüarn et al. reported a 25-year delay [21], and Makol et al. described another patient with two decades of unexplained arthritis [22]. In our case, the patient experienced recurrent polyarthralgia for four years before diagnosis, highlighting how insidious and misleading the disease course can be. 

From a diagnostic perspective, Whipple’s disease is most frequently confirmed by identifying PAS-positive macrophages in affected tissues, most classically duodenal biopsies, but also pericardial, myocardial, synovial, or lymph node samples when the disease is localized [17,18,22], sometimes supported by electron microscopy or PCR [7,16,21]. Histological detection of PAS-positive macrophages or PCR-based identification of *T. whipplei* DNA in tissue samples remains the cornerstone of diagnosis. However, PAS staining lacks specificity, as PAS-positive macrophages may also occur in infections with *M. avium-intracellulare*, *R. equi*, *B. cereus*, *Corynebacterium* spp., *H. capsulatum*, and in other conditions such as Crohn’s disease or histiocytosis [7]. 

Both PAS staining and PCR may yield negative results on intestinal biopsies, while extra-intestinal tissues, particularly myocardial or pericardial samples, can be positive [14,19,23]. Thus, when gastrointestinal biopsies are non-contributory, sampling of cardiac or other extra-intestinal sites should be considered. Notably, the first reported cardiac cases of Whipple’s disease were diagnosed postmortem [23]. Our case reinforces the diagnostic value of pericardial biopsy when intestinal investigations are inconclusive. 

Most reported pericardial cases have required pericardiectomy or pericardial stripping [13,14,16,18,21,22], along with prolonged antibiotic therapy including ceftriaxone, meropenem, doxycycline, or trimethoprim-sulfamethoxazole [13,14,16,21,22]. Standard regimens consist of 14 days of intravenous ceftriaxone or meropenem, followed by one year of oral trimethoprim-sulfamethoxazole, with cure rates approaching 98%. A recent phase 2/3 randomized trial demonstrated non-inferiority of a fully oral doxycycline-hydroxychloroquine regimen [26].

To our knowledge, our patient represents the first reported case of constrictive myopericarditis due to *T. whipplei* successfully treated without pericardiectomy. Given her prohibitive operative risk and rapid clinical and imaging improvement under corticosteroids combined with antibiotics, medical therapy alone proved sufficient. This suggests that in selected patients with potentially reversible inflammatory constriction, a conservative approach may be a valid alternative.

Despite adequate antimicrobial therapy, Whipple’s disease may evolve with significant morbidity or paradoxical inflammatory worsening due to immune reconstitution inflammatory syndrome (IRIS), particularly in patients previously treated with immunosuppressive agents [24]. IRIS typically occurs within weeks to months of initiating antibiotics and manifests as renewed inflammation, arthralgia, serositis, or worsening heart failure despite decreasing microbial burden [24,27]. Mechanistically, IRIS reflects an abrupt shift from the Th2/Treg-dominant, anergic immune profile of untreated Whipple’s disease toward an exaggerated Th1-driven cytokine response involving TNF-α, IFN-γ, and markers of microbial translocation owing to intestinal barrier dysfunction. Corticosteroids are the mainstay of treatment, with thalidomide suggested for refractory cases [27].

In our patient, clinical deterioration after antibiotic initiation, diffuse serositis, persistent cachexia despite microbiologic control, and striking improvement under corticosteroids raised suspicion of a partial IRIS-like phenomenon, although the absence of fever and timing of PCR testing limit definitive classification.

Whipple’s disease is increasingly understood as a consequence of a subtle but profound disequilibrium in host immunity rather than a solely opportunistic infection. Although *T. whipplei* is ubiquitous, only a minority of exposed individuals develop disease, indicating that host-related factors, genetic, innate, and adaptive, play a decisive role [15]. Multiple studies demonstrate defects at the interface of innate and adaptive immunity. Impaired macrophage bactericidal activity, reduced CD11b and iNOS expression, and an M2-polarized macrophage profile that favors intracellular persistence. Diminished IL-12 production and impaired dendritic cell maturation further blunt Th1 responses, resulting in inadequate interferon gamma (IFN-γ)-mediated intracellular pathogen clearance [15,24]. Concomitant increases in IL-16, HLA-G, and type I interferon responses reinforce an immunoregulatory environment characterized by increased apoptosis, impaired antigen presentation, and expansion of regulatory T cells. On the adaptive side, reduced CD4⁺ T-cell counts, inverted CD4/CD8 ratios, weakened cellular immunity, and diminished switched memory B-cell compartments have been documented and likely contribute to susceptibility and dissemination [15].

Within this framework, our patient’s hematologic findings are noteworthy. The presence of a markedly abnormal κ/λ ratio and a small monotypic CD19⁺CD5⁻CD20⁺ κ-restricted B-cell population consistent with non-CLL monoclonal B-cell lymphocytosis is unlikely to be entirely incidental. Although monoclonal B-cell lymphocytosis is typically asymptomatic and age-related, its coexistence with disseminated Whipple’s disease raises the possibility that even mild or subclinical disturbances in B-cell homeostasis may contribute to impaired humoral signaling or suboptimal antigen-driven T-cell activation. Given that B-cell abnormalities and reduced memory B-cell fractions have been described in Whipple’s disease, this clonal population may represent a pre-existing immunologic vulnerability. While causality cannot be inferred, this finding aligns with the broader context of immune dysregulation that permitted *T. whipplei* persistence and atypical organ involvement in our patient.

## Conclusions

Whipple’s disease, although rare, should be considered in cases of unexplained constrictive pericarditis or myopericarditis, especially when accompanied by systemic symptoms such as chronic arthralgia or weight loss. Because gastrointestinal biopsies may be negative in localized forms, cardiac tissue analysis, including PAS staining and PCR, can be essential for diagnosis. Our case is notable as the first report of constrictive myopericarditis due to *T. whipplei *successfully managed without pericardiectomy, suggesting that a purely medical approach may be appropriate in selected high-risk patients when the constriction is predominantly inflammatory. A deeper understanding of the immunopathogenesis, including macrophage dysfunction, impaired Th1 responses, and the risk of IRIS, will be crucial for improving recognition, guiding treatment strategies, and reducing complications.
